# Supplementary material for: Avirulence depletion assay: Combining R gene-mediated selection with bulk sequencing for rapid avirulence gene identification in wheat powdery mildew
Source: PLoS Pathog. 2025 Jan 7;21(1):e1012799. doi: 10.1371/journal.ppat.1012799 (PMC11741615; doi:10.1371/journal.ppat.1012799)
Supplement: S1 Note — (DOCX) [file ppat.1012799.s001.docx]

**S1 Note: PacBio HiFi-based telomere-to-telomere assembly of Swiss *Bgt* isolate CHVD_042201**

To create a *de novo* assembly of *Bgt* isolate CHVD_042201 we sequenced its genome to a coverage of approximately 100X using CCS HiFi reads on the PacBio Sequel II system. Using the resulting 12.8M PacBio CCS HiFi reads, we first benchmarked three commonly used genome assemblers specifically designed for HiFi reads: HiFlye (1), HiCanu (2), and hifiasm (3). To assess the quality of the resulting assemblies, we performed whole genome alignments against the *Bgt* reference genome (Bgt_genome_v3_16, (4)), which has assembled chromosomes that were resolved based on information of a genetic map and BAC-end sequences, and subsequently evaluated the ability of each assembler to resolve chromosome structures and complex regions such as centromeres. Notably, the use of the hifiasm assembler resulted in better resolution of both chromosomes and centromeres. Hifiasm successfully assembled seven out of the eleven chromosomes completely, compared to five for HiCanu and two for HiFlye. Additionally, all eleven centromeric regions were assembled without gaps in the hifiasm assembly, whereas HiCanu and HiFlye only assembled nine and six centromeres, respectively. Consequently, we chose to use the hifiasm assembler for all subsequent analyses.

Investigating the regions causing alignment breaks in the hifiasm assembly revealed that they corresponded to two rDNA clusters located on Chr-05 (5.8S rDNA) and Chr-09 (18S/28S rDNA), a tandem repeat cluster specific to *Blumeria* on Chr-04 (4), and a candidate effector cluster on Chr-01. Strikingly, all these regions shared a common characteristic: due to their repetitive nature, they exhibited higher read coverage than the remaining regions of the genome. We hypothesized that subsampling of sequencing reads might improve the assembly resolution of these complex regions. Indeed, subsampling to approximately 30, 40, 50, and 60X coverage improved the assembly of the effector cluster on Chr-01 as well as the 5.8S rDNA cluster on Chr-05 for multiple subassemblies. For instance, the alignment break on Chr-01 was resolved in 9 out of the 20 subassemblies. Similarly, the rDNA cluster on Chr-05 was assembled in 4 out of 20 subassemblies. However, the tandem repeat cluster on Chr-04 and the rDNA cluster on Chr-09 were not resolved in any of the subassemblies.

To create the final CHVD_042201 assembly, we utilized the initially produced hifiasm assembly based on all sequencing reads. Subsequently, we filled above-mentioned gaps with the corresponding sequences originating from one of the subsampled assemblies. For the effector cluster on Chr-01 all 9 subassemblies that resolved the cluster were identical, thus a random one was chosen to fill the gap. For the 5.8S rDNA cluster on Chr-05 the assembly with the longest repeat cluster was selected. This strategy allowed us to assemble nine of the eleven *Bgt* chromosome gapless (i.e. telomere-to-telomere), with Chr-09 and Chr-04 each containing a single remaining sequence gap corresponding to the 18S/28S rDNA cluster and the tandem repeat cluster, respectively.

Hifiasm initially also failed to assemble the mitochondrial genome, likely due to the fact that sequence coverage of the mitochondrion was exceeding the coverage of the nuclear genome. In the primary assembly, 275 contigs corresponded to the mitochondrial genome. We removed these contigs from the initial assembly, and identified all HiFi reads corresponding to the mitochondrion by aligning the reads to previously published mitochondrial genomes of isolates CHE_96224 using BLAST. Subsequently, we used hifiasm to separately assemble all sequencing reads corresponding to the mitochondrial genome, which resulted in a single circular contig of 102.7 kb, as well as 16 short contigs that were not incorporated into the final assembly. We also removed 157 contigs that contained additional sequences corresponding to rDNA clusters and five contigs that had BLAST hits to the genus *Penicillium*, likely indicating contamination. After one round of polishing the assembly using short-read DNA sequencing of isolate CHVD_042201 with the Pilon software (5), the final assembly of CHVD_042201 consisted of 141.2 Mb, resolved into eleven *Bgt* chromosomes, a 100 kb mitochondrial genome, and five short unassembled contigs. We found 22 occurrences of the telomere repeat sequence at the ends of the eleven chromosomes of CHVD_042201.

Alignment of short-read DNA sequences of CHVD_042201 against the final genome assembly, followed by read coverage analysis in 500 bp windows, indicated that only 446 kb of the assembly sequence might still contain collapsed sequences. This is a significant improvement compared to the 2.24 Mb of collapsed sequence in the *Bgt* reference genome Bgt_genome_v3_16 of isolate CHE_96224 (4). In the CHVD_042201 assembly, the collapsed regions are mostly confined to the highly repetitive tandem duplicated rDNA cluster and the tandem repeat cluster on Chr-04. We estimated this collapsed region to account for an additional 5.6 Mb of sequences, bringing the total size of the CHVD_042201 genome to an estimated 147.9 Mb (S2 Table).

Finally, we used the maker2 software to create a homology-based draft annotation for CHVD_042201. We used the predicted proteomes of both *Bgt* reference genome CHE_96224 and *Blumeria hordei* reference genome DH14 (6) to predict gene models in CHVD_042201. This strategy resulted in a draft genome annotation of 9’932 genes in the Bgt_CHVD042201_genome_v1.

The genome assembly of CHVD_042201 has been deposited in the European Nucleotide Archive (ENA) under accession number GCA_964289775. Additionally, genome assembly and annotation are available from Zenodo: <https://zenodo.org/records/11233413> .

**References**

1. Kolmogorov M, Yuan J, Lin Y, Pevzner PA. Assembly of long, error-prone reads using repeat graphs. Nature Biotechnology. 2019;37(5):540-+.

2. Nurk S, Walenz BP, Rhie A, Vollger MR, Logsdon GA, Grothe R, et al. HiCanu: accurate assembly of segmental duplications, satellites, and allelic variants from high-fidelity long reads. Genome Research. 2020;30(9):1291-305.

3. Cheng HY, Concepcion GT, Feng XW, Zhang HW, Li H. Haplotype-resolved de novo assembly using phased assembly graphs with hifiasm. Nature Methods. 2021;18(2):170-+.

4. Müller MC, Praz CR, Sotiropoulos AG, Menardo F, Kunz L, Schudel S, et al. A chromosome-scale genome assembly reveals a highly dynamic effector repertoire of wheat powdery mildew. New Phytologist. 2019;221(4):2176-89.

5. Walker BJ, Abeel T, Shea T, Priest M, Abouelliel A, Sakthikumar S, et al. Pilon: An integrated tool for comprehensive microbial variant detection and genome assembly improvement. Plos One. 2014;9(11):e112963.

6. Frantzeskakis L, Kracher B, Kusch S, Yoshikawa-Maekawa M, Bauer S, Pedersen C, et al. Signatures of host specialization and a recent transposable element burst in the dynamic one-speed genome of the fungal barley powdery mildew pathogen. Bmc Genomics. 2018;19:381.
