# Supplementary material for: Avirulence depletion assay: Combining R gene-mediated selection with bulk sequencing for rapid avirulence gene identification in wheat powdery mildew
Source: PLoS Pathog. 2025 Jan 7;21(1):e1012799. doi: 10.1371/journal.ppat.1012799 (PMC11741615; doi:10.1371/journal.ppat.1012799)
Supplement: S2 Note — (DOCX) [file ppat.1012799.s002.docx]

**S2 Note: Performing the AD assay based on alternative reference genomes**

In this study, we developed a bioinformatics pipeline to analyze data generated by the AD assay and identify regions showing deviation from a 1:1 parental genotype ratio in experimentally generated bulk sequencing data in the haploid organism *Bgt*. The experimental setup in this study allowed us to perform our analysis based on the novel high-quality reference genome of CHVD_042201, which represented the avirulent parental isolate on *Pm3a* and *Pm60* resistance genes under investigation. However, this particular experimental setup might not always be achievable due to the significant cost and time investment required to create a high-quality genome assembly of *Bgt.*

For *Bgt*, four high-quality genome assemblies are publicly available, including the CHVD_042201 assembly presented in this study (1-3). Our goal was to develop the pipeline for the AD assay to run on any *Bgt* reference genome, in order to maximize the success of *AVR* gene identification even without a high-quality reference genome of the avirulent isolate used for the genetic cross. Therefore, we tested our pipeline using the *Bgt* reference genome assembly of isolate CHE_96224 (Bgt_genome_v3_16) as the basis of the AD assay and compared the results with the initial analysis relying on the CHVD_042201 genome assembly.

In a first step, we mapped the Illumina sequencing data of both parental isolates (CHVD_042201, CHN_52_27) and the selected and unselected bulks against the reference assembly Bgt_genome_v3_16. Similar to our initial analysis using the CHVD_042201 genome, we then proceeded to call SNPs between the two parental isolates CHVD_042201 and CHN_52_27 in order to use them as genetic markers for the analysis of the bulks. Based on this strategy, we identified 193’871 SNP markers segregating between CHVD_042201 and CHN_52_27 when using the Bgt_genome_v3_16, compared to 198’027 SNP markers identified when using the Bgt_CHVD042201_genome_v1 as a reference, showing that the number of markers identified with both approaches is comparable.

The subsequent genotype depletion analysis, using the G-test to identify deviations from the parental genotype ratio of 1:1, was consistent across the analyses with either of the two reference assemblies. Both approaches identified a single region on *Bgt* Chr-06 for the *Pm3a*- and *Pm60*-selected bulks, which was absent in the non-selected bulk created on the susceptible cultivar ‘Kanzler’ (S1 Fig, S2 Table). This demonstrates that the AD pipeline is effective on an alternative reference genome assembly that does not represent the avirulent parental isolate used for the genetic cross and is therefore broadly applicable.

We analyzed the regions showing the strongest depletion signal (i.e. 95% and 90% of reads originating from the virulent parent) from both analyses. For *AvrPm3^a2/f2^*, the interval based on Bgt_genome_v3_16 was 274 kb, significantly larger than the 25 kb interval identified using the CHVD_042201 assembly. In contrast, the interval for *AvrPm60* was 535 kb based on Bgt_genome_v3_16 compared to the 667 kb interval identified in our initial analysis based on the CHVD_042201 assembly. In both cases, the *AvrPm3^a2/f2^* and *AvrPm60* genes were located within the identified genomic regions, thus showing that the AD assay identified the correct genomic location.

In summary, we demonstrate that the AD assay successfully identifies the *AvrPm3^a2/f2^* and *AvrPm60* loci when Bgt_genome_v3_16 is used as the reference genome, suggesting that the AD pipeline can readily be used with other *Bgt* reference genomes. However, we recommend using a reference genome from an avirulent isolate on the *R* gene under investigation in order to ensure efficient avirulence candidate gene definition. This is best exemplified by the case of the *AvrPm60* genes, where the identified virulence allele in isolate CHN_52_27 is defined by a large-scale deletion encompassing of all three *AvrPm60* copies (Fig 3c). In such a scenario, the use of a reference genome from a virulent strain carrying the same deletion would fail to identify *AvrPm60* candidate genes.

**References**

1. Müller MC, Kunz L, Graf J, Schudel S, Keller B. Host adaptation through hybridization: Genome analysis of triticale powdery mildew reveals unique combination of lineage-specific effectors. bioRxiv. 2021:2021.05.06.442769.

2. Müller MC, Kunz L, Schudel S, Lawson AW, Kammerecker S, Isaksson J, et al. Ancient variation of the AvrPm17 gene in powdery mildew limits the effectiveness of the introgressed rye Pm17 resistance gene in wheat. Proceedings of the National Academy of Sciences of the United States of America. 2022;119(30):e2108808119-e.

3. Müller MC, Praz CR, Sotiropoulos AG, Menardo F, Kunz L, Schudel S, et al. A chromosome-scale genome assembly reveals a highly dynamic effector repertoire of wheat powdery mildew. New Phytologist. 2019;221(4):2176-89.
