# Supplementary figures and images for: Avirulence depletion assay: Combining R gene-mediated selection with bulk sequencing for rapid avirulence gene identification in wheat powdery mildew
Source: PLoS Pathog. 2025 Jan 7;21(1):e1012799. doi: 10.1371/journal.ppat.1012799 (PMC11741615; doi:10.1371/journal.ppat.1012799)

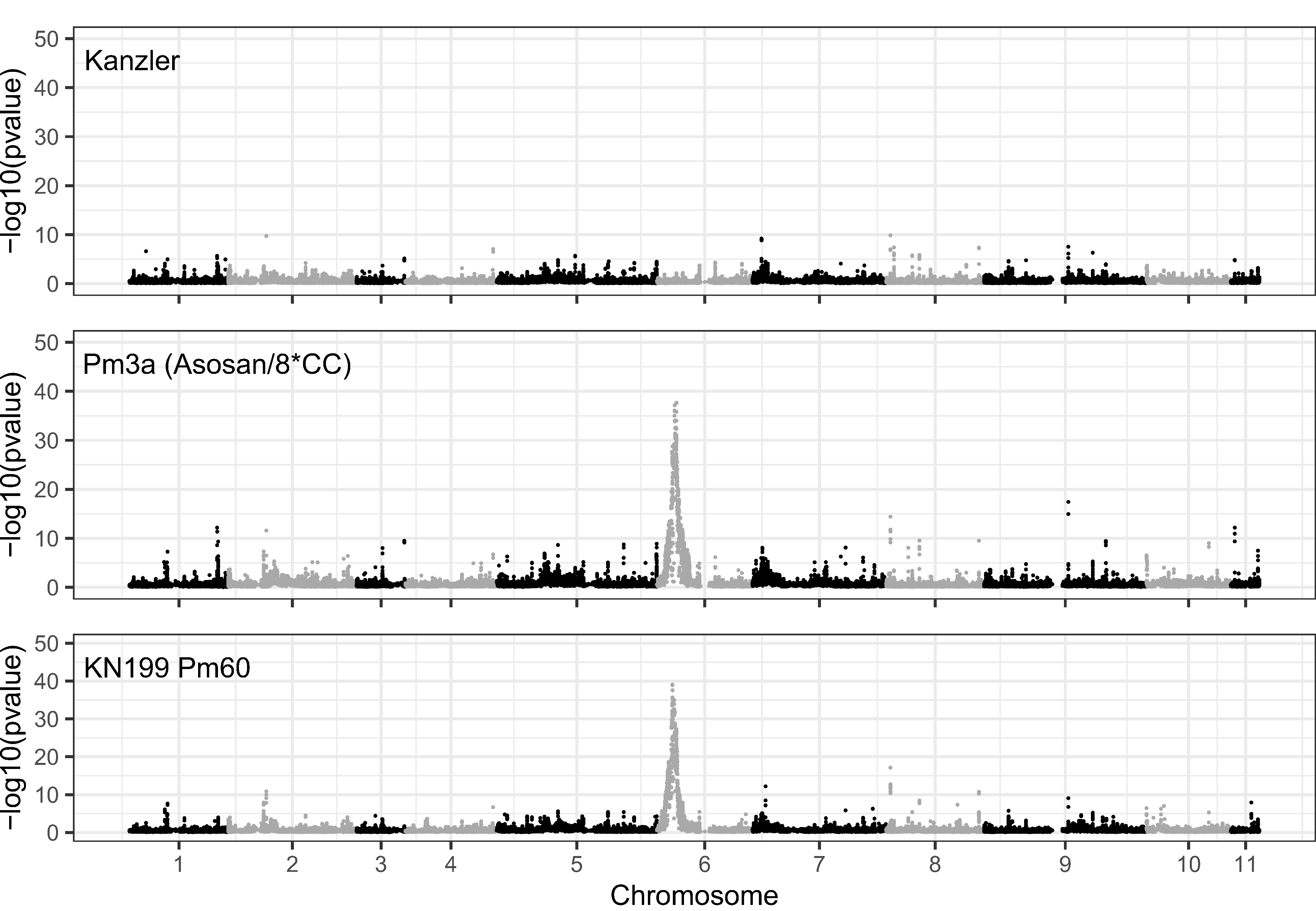

Supplement: S1 Fig — The AD assay based on the CHVD_042201 x CHN_52_27 cross was performed using the reference genome of Bgt_genome_v3_16 (CHE_96224). Cultivars used for selection are indicated in the respective plots. The Y-axis indicates–log10(p-values) of G-test statistics used to test for a deviation from 1:1 parental genotype ratio expected in the absence of selection. G-test values were averaged over 10 neighbouring SNPs plotted along the 11 chromosomes of Bgt isolate CHE_96224 (x-axis). (TIFF) [file ppat.1012799.s003.tiff]

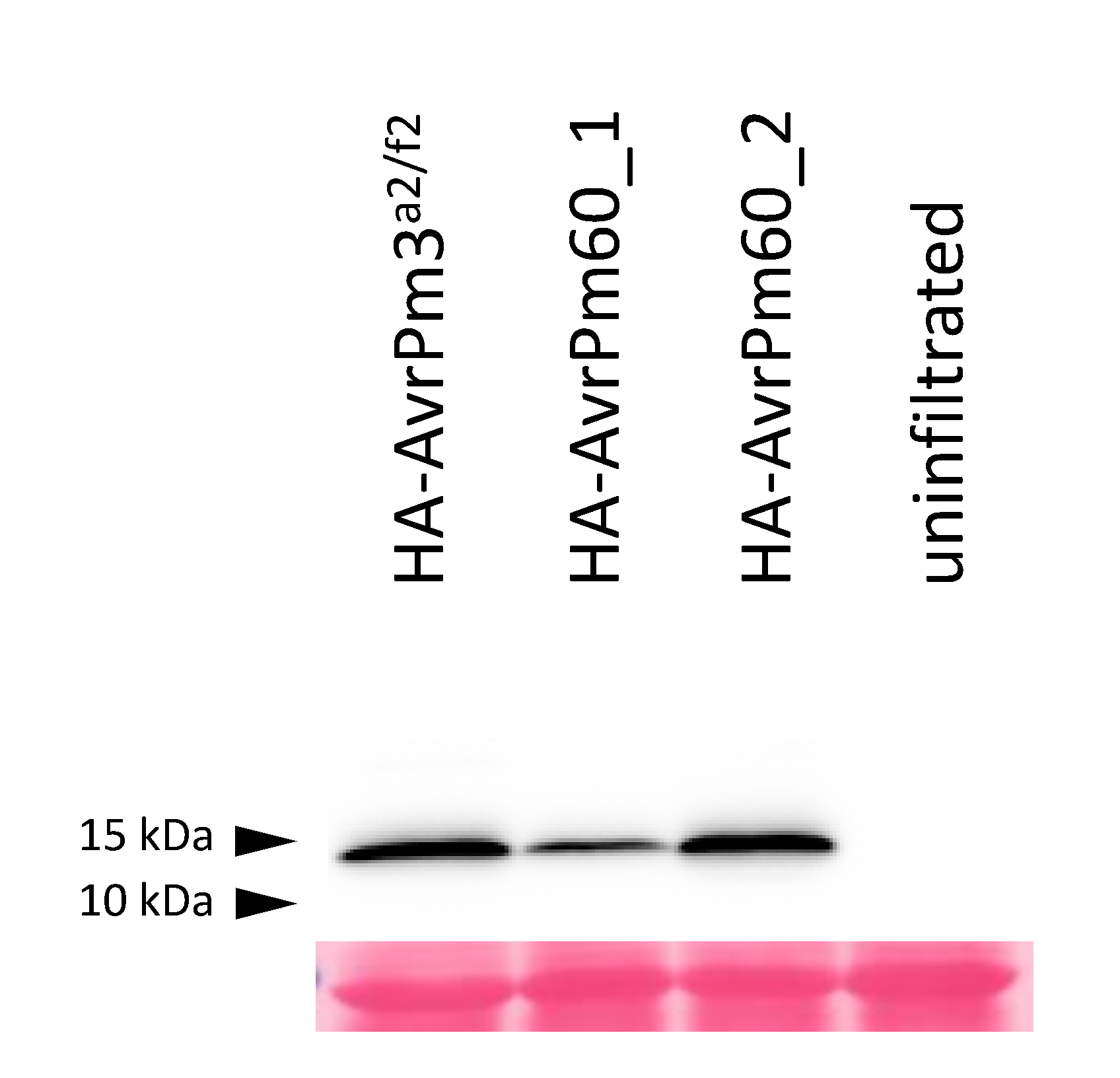

Supplement: S2 Fig — Detection of HA-AvrPm60_1 and HA-AvrPm60_2 by anti-HA western blotting (top panel). HA-AvrPm3a2/f2 served as a positive control, protein extracts from uninfiltrated N. benthamiana leaf areas served as a negative control. Total protein Ponceau S staining is shown as a loading control (bottom panel). Black arrows indicate 10 kDa and 15 kDa protein size markers. Protein expression, protein extraction and Western blot analysis were performed three times with similar results. (TIFF) [file ppat.1012799.s004.tiff]

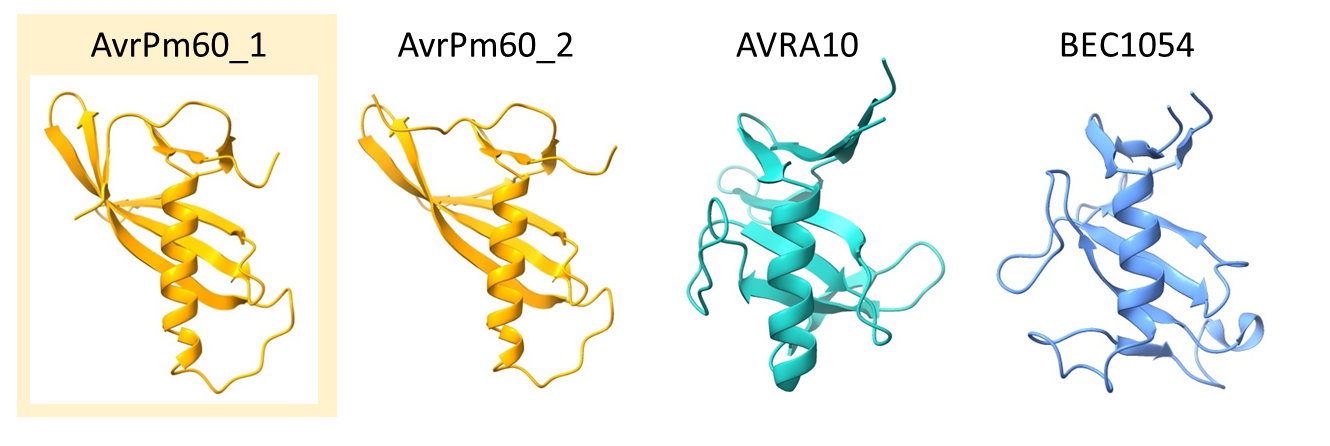

Supplement: S3 Fig — The predicted three-dimensional structures of AvrPm60_1 and AvrPm60_2, according to Alphafold 3 structural modelling, are shown. The predicted AvrPm60_1 structure, shown in Fig 3E, is highlighted in yellow. The experimentally validated structures of AVRA10 (8OXK) [29] and BEC1054 (6FMB) [37] are shown as comparison. (TIFF) [file ppat.1012799.s005.tiff]

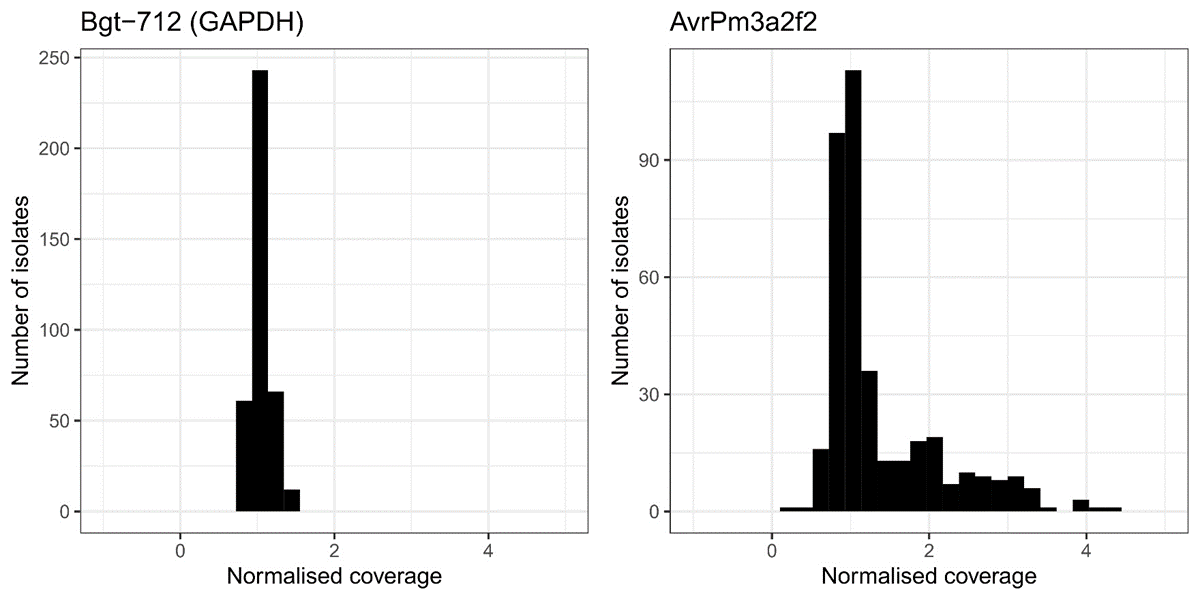

Supplement: S5 Fig — For each gene, the genomic coverage of sequencing reads was normalised to the coverage of all genes in the genome. (TIFF) [file ppat.1012799.s007.tiff]

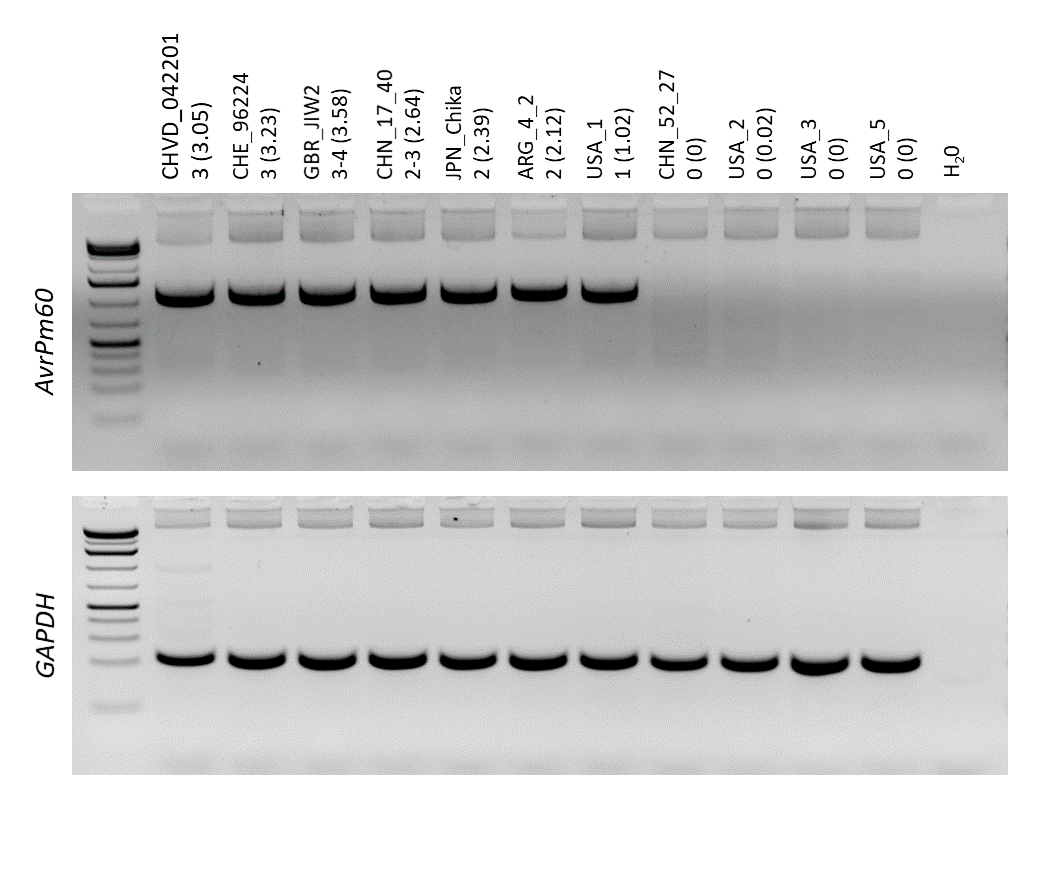

Supplement: S6 Fig — AvrPm60 specific primers were designed in conserved regions flanking the three AvrPm60 genes (CHVD042201-04743, CHVD042201-04745 and CHVD042201-04747) in CHVD_042201 with a predicted amplicon size of 1179bp. Fungal GAPDH served as a positive control. The estimated number of AvrPm60 gene copies according to sequencing coverage analysis are indicated next to the isolate name with normalized sequencing coverage indicated in brackets. The isolates CHVD_042201 and CHN_52_27 used for initial AvrPm60 identification are shown as comparison. (TIFF) [file ppat.1012799.s008.tiff]

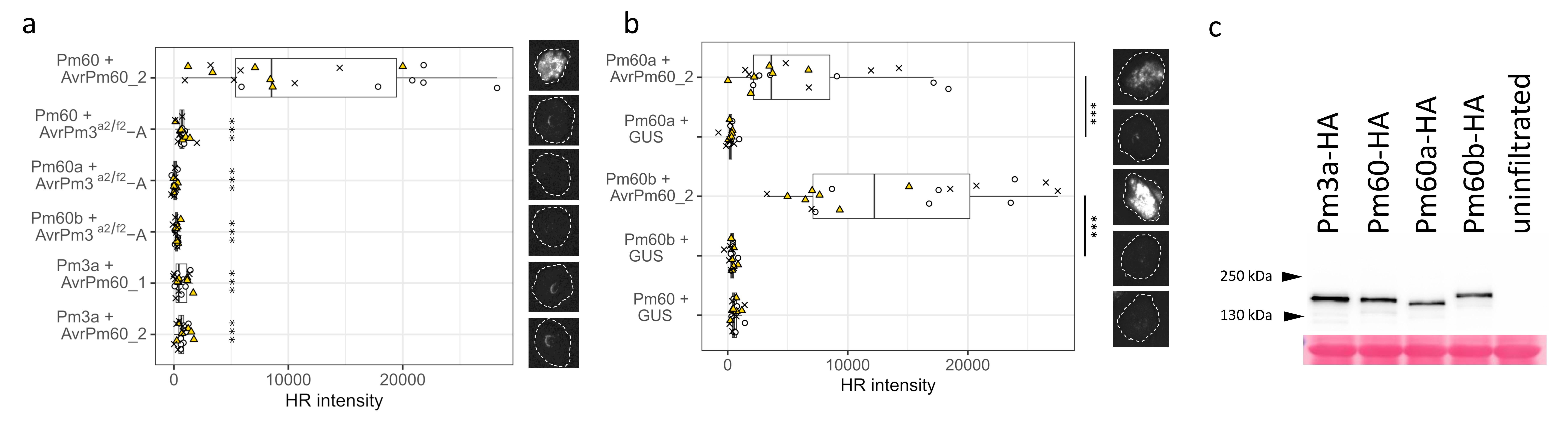

Supplement: S7 Fig — (a) Agrobacterium-mediated co-expression of Pm60, Pm60a and Pm60b with the non-corresponding AVR effector AvrPm3a2/f2, and co-expression of AvrPm60_1 and AvrPm60_2 with the non-corresponding NLR Pm3a does not result in a detectable HR response. Co-expression of AvrPm60_2 with Pm60 served as a positive control. Asterisks above the boxplots indicate statistical differences compared to the positive control according to post-hoc pairwise Wilcoxon rank sum exact tests (* p<0.05, ** p< 0.01, *** p< 0.001) performed after a significant Kruskal-Wallis test (p<0.05). P-values were adjusted to account for multiple comparison using the Benjamini & Hochberg method. (b) Pm60, Pm60a and Pm60b do not exhibit autoactivity upon co-expression with GUS. Co-expressions of AvrPm60_2 with Pm60a or Pm60b served as positive controls. Co-infiltrations in (a) and (b) were performed with a 4(effector): 1 (NLR) ratio. Leaves were imaged at 4dpi using the Fusion FX imager system. The assay was performed with n = 6 leaves and repeated a total of three times with similar results (total n = 18 leaves). Boxplots represent quantification of HR intensity in the N. benthamiana expression assay. Datapoints from the three independent experiments are indicated by symbols (cross, circle, triangle) and additionally color coded. Asterisks indicate statistical differences according to post-hoc pairwise Wilcoxon rank sum tests (* p<0.05, ** p< 0.01, *** p< 0.001) performed after a significant Kruskal-Wallis test (p<0.05). P-values were adjusted to account for multiple comparison using the Benjamini & Hochberg method. (c) Detection of Pm60-HA, Pm60a-HA and Pm60b-HA expressed in N.benthamiana by anti-HA western blotting (top panel). Pm3a-HA served as a positive control, protein extracts from uninfiltrated N. benthamiana leaf areas served as a negative control. Total protein Ponceau S staining is shown as a loading control (bottom panel). Black arrows indicate 130 kDa and 250 kDa protein size markers. Protein ex [file ppat.1012799.s009.tiff]
