# Supplementary material for: Avirulence depletion assay: Combining R gene-mediated selection with bulk sequencing for rapid avirulence gene identification in wheat powdery mildew
Source: PLoS Pathog. 2025 Jan 7;21(1):e1012799. doi: 10.1371/journal.ppat.1012799 (PMC11741615; doi:10.1371/journal.ppat.1012799)
Supplement: S1 Table — (DOCX) [file ppat.1012799.s010.docx]

**S1 Table: Genome stastistics of Bgt_CHVD042201_genome_v1**

| *Bgt* isolate | CHVD_042201 | CHE_96224 |
| --- | --- | --- |
| Assembly | Bgt_CHVD_042201_genome_v1 | Bgt_genome_v3_16^a^ |
| Assembly size | 141'167'024 | 140'575'254 |
| Largest scaffold | 16'394'915 | 4'473'759 |
| Number of sequence gaps | 2 | 357 |
| Chromosome number | 11 | 11 |
| Telomeric repeats | 22 | 7 |
| Number of genes | 9’932 | 8’581 |

^a^ see (1)

**Reference**

1. Müller MC, Praz CR, Sotiropoulos AG, Menardo F, Kunz L, Schudel S, et al. A

chromosome-scale genome assembly reveals a highly dynamic effector repertoire of wheat

powdery mildew. New Phytologist. 2019;221(4):2176-89.
