# Supplementary material for: Avirulence depletion assay: Combining R gene-mediated selection with bulk sequencing for rapid avirulence gene identification in wheat powdery mildew
Source: PLoS Pathog. 2025 Jan 7;21(1):e1012799. doi: 10.1371/journal.ppat.1012799 (PMC11741615; doi:10.1371/journal.ppat.1012799)
Supplement: S2 Table — (DOCX) [file ppat.1012799.s011.docx]

**S2 Table: Results of the AD assay using different reference genomes**

| **Reference genome** | **Cross** | **Selection line** | **Selection strength** | **Start** | **End** | **Interval size** |
| --- | --- | --- | --- | --- | --- | --- |
| Bgt_CHVD042201_genome_v1 | CHVD_042201 x CHN_52_27 | Asosan/8*CC | 90% | 1911738 | 2647215 | 735477 |
| Bgt_CHVD042201_genome_v1 | CHVD_042201 x CHN_52_27 | Asosan/8*CC | 95% | 2272209 | 2297683 | 25474 |
| Bgt_CHVD042201_genome_v1 | CHVD_042201 x CHN_52_27 | Kn199 Pm60 | 90% | 1765214 | 2701804 | 936590 |
| Bgt_CHVD042201_genome_v1 | CHVD_042201 x CHN_52_27 | Kn199 Pm60 | 95% | 1904112 | 2570698 | 666586 |
| Bgt_genome_v3_16 | CHVD_042201 x CHN_52_27 | Asosan/8*CC | 90% | 1842969 | 2463023 | 620054 |
| Bgt_genome_v3_16 | CHVD_042201 x CHN_52_27 | Asosan/8*CC | 95% | 2101179 | 2375031 | 273852 |
| Bgt_genome_v3_16 | CHVD_042201 x CHN_52_27 | Kn199 Pm60 | 90% | 1369058 | 2483455 | 1114397 |
| Bgt_genome_v3_16 | CHVD_042201 x CHN_52_27 | Kn199 Pm60 | 95% | 1839679 | 2375034 | 535355 |
