# Supplementary material for: Avirulence depletion assay: Combining R gene-mediated selection with bulk sequencing for rapid avirulence gene identification in wheat powdery mildew
Source: PLoS Pathog. 2025 Jan 7;21(1):e1012799. doi: 10.1371/journal.ppat.1012799 (PMC11741615; doi:10.1371/journal.ppat.1012799)
Supplement: S3 Table — (DOCX) [file ppat.1012799.s012.docx]

**S3 Table: List of primers used in this study.**

| **Name** | **Sequence (5'-3')** | **Description/Purpose** |
| --- | --- | --- |
| HZ108 | GGGGACAAGTTTGTACAAAAAAGCAGGCTTCATGGAATCGGCGATTGGCGCG | BP cloning to generate pDONR221-Pm60 |
| HZ109 | GGGGACCACTTTGTACAAGAAAGCTGGGTCCTACTCAAGTTCAAGTATCACATT | BP cloning to generate pDONR221-Pm60 |
| LK1102 | GAATGTGGCAACTTCTTTCTG | In-Fusion cloning to generate pDONR221-Pm60a |
| LK1103 | GGAAAAGCTTGATGGTGTG | In-Fusion cloning to generate pDONR221-Pm60a |
| LK1075 | ATCAGTTACAGTAAAAGAATGTGG | In-Fusion cloning to generate pDONR221-Pm60b |
| LK1076 | CAGAGCCATTGACTGCATG | In-Fusion cloning to generate pDONR221-Pm60b |
| LK1144 | CCAGATTATGCTTGAGACCCAGCTTTCTTGTACAAAG | Site-directed mutagenesis to introduce C-terminal HA tag on Pm60, Pm60a and Pm60b |
| LK1145 | AACATCGTATGGATACTCAAGTTCAAGTATCACATTAAAATCTG | Site-directed mutagenesis to introduce C-terminal HA tag on Pm60, Pm60a and Pm60b |
| LK1146 | GTTCCAGATTACGCTGAAGGTAATTGCAATTACAAATGCG | Site-directed mutagenesis to introduce N-terminal HA tag on AvrPm60_1 and AvrPm60_2 |
| LK1147 | ATCGTATGGGTACATGGTGAAGGGGGCGG | Site-directed mutagenesis to introduce N-terminal HA tag on AvrPm60_1 and AvrPm60_2 |
| LK1116 | GGATGTTTGGATCGCTC | *AvrPm60* specific primer |
| LK1117 | GTATATAGCAAAGGCTTGATG | *AvrPm60* specific primer |
| GAPDH_F | TGTCTTCCGAAACGCTGCTC | GAPDH specific primer (Bourras et al., 2015, 10.1105/tpc.15.00171) |
| GAPDH_R | AGTCCGTCCTCGACTGCTTGT | GAPDH specific primer (Bourras et al., 2015, 10.1105/tpc.15.00171) |
