# Supplementary material for: Avirulence depletion assay: Combining R gene-mediated selection with bulk sequencing for rapid avirulence gene identification in wheat powdery mildew
Source: PLoS Pathog. 2025 Jan 7;21(1):e1012799. doi: 10.1371/journal.ppat.1012799 (PMC11741615; doi:10.1371/journal.ppat.1012799)
Supplement: S4 Table — (DOCX) [file ppat.1012799.s013.docx]

**S4 Table: Sequences of DNA fragments produced by gene synthesis.**

| **Name** | **Sequence (5'-3')** |
| --- | --- |
| CHVD042201-04754  (AvrPm3^a2/f2^-A) | ATGGGTCCTGTCGCAAATGCTAGTTCTTATAAATGTCACGATAGAGTCATTGGTCCAGTGACTCTGAATGACCAGATCGAGAAGGCTTACCGTGAGGCCCTGGAGGCCGGCACGTCACCAAATGGACTTAGGAAAGGCCAGAAATTCGGTTCTCGTTATTTCAACGTGATTCTTAAGAGGGGTGAGGAGAACATTAAAGTTGAATTCTTTGTTGGTATTAACTATCTCAAAGAGATTATTTACCTTCAGGCATATGTCCAGAGCGTTTTACTTGACTGTTACCCCACGACCGAGCGACCACAGTTGAACATTATCTTGCACTAA |
| CHVD042201-04754_CHN52-27 (H36Q_G84E_E121D)  (AvrPm3^a2/f2^-B) | ATGGGTCCTGTCGCAAATGCTAGTTCTTATAAATGTCAAGATAGAGTCATTGGTCCAGTGACTCTGAATGACCAGATCGAGAAGGCTTACCGTGAGGCCCTGGAGGCCGGCACGTCACCAAATGGACTTAGGAAAGGCCAGAAATTCGGTTCTCGTTATTTCAACGTGATTCTTAAGAGGGAAGAGGAGAACATTAAAGTTGAATTCTTTGTTGGTATTAACTATCTCAAAGAGATTATTTACCTTCAGGCATATGTCCAGAGCGTTTTACTTGACTGTTACCCCACGACCGATCGACCACAGTTGAACATTATCTTGCACTAA |
| CHVD042201-04766 | ATGGACATTTCCAACTACTTGTGTGACCACGTCGTCTTGGACGCAAAGGACATAGAGGCCGGAGTTGATAGAGCCTTTAGAACTAAAATGCAAGAGACGTTGGGGGCCTACGCCCCTGACGATTTCTATAACGAGGGCTCATATATCGTTAAGTATAAGTCTCCAAGGGTTAATATGGATGTCACTATTAAAATTGGTATCACGTTTAGTGAAGACGTACTTTACGTCAAAGCCGCAGGTGATGGGCAGGAGATAGATTGTCACCCGACCGACAAACCGGCCACGACGAAACGTATCGTCCCGTAA |
| CHVD042201-04766_CHN52-27 (Y73N) | ATGGACATTTCCAACTACTTGTGTGACCACGTCGTCTTGGACGCAAAGGACATAGAGGCCGGAGTTGATAGAGCCTTTAGAACTAAAATGCAAGAGACGTTGGGGGCCTACGCCCCTGACGATTTCTATAACGAGGGCTCATATATCGTTAAGAATAAGTCTCCAAGGGTTAATATGGATGTCACTATTAAAATTGGTATCACGTTTAGTGAAGACGTACTTTACGTCAAAGCCGCAGGTGATGGGCAGGAGATAGATTGTCACCCGACCGACAAACCGGCCACGACGAAACGTATCGTCCCGTAA |
| CHVD042201-04767 | ATGGAGTTTTCTAACTATTTATGCGACCATATCGTAATAAATAAGAAAGACATAGAGTATTCAGTTGACCATGCTTTCAAAAAGCGTATGCAGGCTAACTTAGGAAAGTTCAAACCCGACGAAAAGTTCGGGACTGCTGGTTATATCACGAAATACAAGACGGAGAAGGAAATTTTCGACGTTCATATTATTATCGAATATACAATCAACGAAGAAGTGATATCTGTTATCGCCAAAGGGCGTGGGCAGCAGGTGGTTTGCCACCCCACAGATCAGCCGGCCACGGAATATACAGAAGTAAGCGGGTCAGGCTAA |
| CHVD042201-04767_CHN52-27 (K74STOP) | ATGGAGTTTTCTAACTATTTATGCGACCATATCGTAATAAATAAGAAAGACATAGAGTATTCAGTTGACCATGCTTTCAAAAAGCGTATGCAGGCTAACTTAGGAAAGTTCAAACCCGACGAAAAGTTCGGGACTGCTGGTTATATCACGAAATACTAA |
| CHVD042201-04752 | ATGTATTCTCCAGTCCCTGTCGCCGAAGCCAGCAGTTATAAATGCCAAGATCGTGTAATTGGTCCTGTGACCCTTAACGATCAGATCAATAAGGCATACGCCGAGGCACAGAGTAATCAATCACGTGGACTGACCCGAGAGCAAATCTTTGCTTCCCGTCAATTTAGGGTAATCTTGACGAGAGACGGCGAGCGTATCTTAATCCAGTTTTACCTGAGTATAAACAACGTCAAAGAGATTTTGTCCTTGCAAGCATACGTTATGAACCAATTATTCACTTGTCATCCCACGATTGAACCACCACAATTAAACGAGGTTTTGCACTAA |
| CHVD042201-04752_CHN52-27 (I120T) | ATGTATTCTCCAGTCCCTGTCGCCGAAGCCAGCAGTTATAAATGCCAAGATCGTGTAATTGGTCCTGTGACCCTTAACGATCAGATCAATAAGGCATACGCCGAGGCACAGAGTAATCAATCACGTGGACTGACCCGAGAGCAAATCTTTGCTTCCCGTCAATTTAGGGTAATCTTGACGAGAGACGGCGAGCGTATCTTAATCCAGTTTTACCTGAGTATAAACAACGTCAAAGAGATTTTGTCCTTGCAAGCATACGTTATGAACCAATTATTCACTTGTCATCCCACGACCGAACCACCACAATTAAACGAGGTTTTGCACTAA |
| CHVD042201-04743; CHVD042201-04745 (AvrPm60_1) | ATGGAAGGTAATTGCAATTACAAATGCGGTCCAGCAGTTATTGATGGTGATTATGTCAGGGAATGTGTCAAGTCTTACTACGAATATAAGATGCGTACCATAGATAGGGATTACGGTCCCAACGATCATTTCACCACCGTCACCTTTCCGTTACAATACTTGCACAAGGAGGAGATCATAACCGTACAAGTCAGCGCCGATTTTACGGCACTTAGGGAAATAACCTCAGTTCGAGCAAGTGCTCTGGAGCAAGAGATAGAGTGTCTGCCGACCCAATTGAAGCCTTCCTACGGAAAAGCCACCTGA |
| CHVD042201-04747 (E103G) (AvrPm60_2) | ATGGAAGGTAATTGCAATTACAAATGCGGTCCAGCAGTTATTGATGGTGATTATGTCAGGGAATGTGTCAAGTCTTACTACGAATATAAGATGCGTACCATAGATAGGGATTACGGTCCCAACGATCATTTCACCACCGTCACCTTTCCGTTACAATACTTGCACAAGGAGGAGATCATAACCGTACAAGTCAGCGCCGATTTTACGGCACTTAGGGAAATAACCTCAGTTCGAGCAAGTGCTCTGGGTCAAGAGATAGAGTGTCTGCCGACCCAATTGAAGCCTTCCTACGGAAAAGCCACCTGA |
| Pm60a_fragment | CACACCATCAAGCTTTTCCCTGCTTCCCTCGAGACACTTGAGATTGAAGGAGAGTCAGGCATGCAGTCAATGGCTCTGCTCAGCAATCTGAAATCCCTAAGGAGACTAGATGTCAGAAGATGCAGCATCACGTGCCATGGACTGCAGGACCTCGCGTGCCTCCAATCAGTTACAGTAAAAGAATGTGGCAACTTCTTTCTG |
| Pm60b_fragment | CATGCAGTCAATGGCTCTGCTCAGCAATCTGAAATCCCTAAGGAGACTAGATGTCAGAAGATGCAGCATCACGTGCCATGGACTGCAGGACCTCGCATGCCTCCAATCACTTACAGTACAAGACTGTGGCAACTTCTTTCCATGGCCTACCGAAGCAGCTCACACCGTCAATCCTTTCCCTCACACCATCAAGCCTTTCCCTGCTTCCCTCGAGACACTTGAGATTGAAGGAGAGTTAGGCATGCAGCCAGTGGCTTTGCTCAGCAATCTGAAATCCCTAAGAAGACTAGATGTCAGAAGATGCAGCATCACGTGCCATGGACTGCAGGACCTCGCATGCCTCCAATCACTTACAGTACAAGACTGTGGCAACTTCTTTCCATGGCCTACCGAAGCAGCTCACACCGTCAATCCTTTCCCTCACACCATCAAGCCTTTCCCTGCTTCCCTCGAGACACTTGAGATTGAAGGAGAGTTAGGCATGCAGCCAGTGGCTTTGCTCAGCAATCTGAAATCCCTAAGAAGACTAGATGTCAGAAGATGCAGCATCACGTGCCATGGACTGCAGGACCTCGCGTGCCTCCAATCAGTTACAGTAAAAGAATGTG |
