# Supplementary material for: Avirulence depletion assay: Combining R gene-mediated selection with bulk sequencing for rapid avirulence gene identification in wheat powdery mildew
Source: PLoS Pathog. 2025 Jan 7;21(1):e1012799. doi: 10.1371/journal.ppat.1012799 (PMC11741615; doi:10.1371/journal.ppat.1012799)
Supplement: S5 Table — (DOCX) [file ppat.1012799.s014.docx]

**S5 Table: RNA sequencing datasets used in this study.**

| **Isolate** | **Type** | **Accession number** | **Publication** |
| --- | --- | --- | --- |
| CHE_96224 rep1 | RNAseq | SRX3503528 | (1) |
| CHE_96224 rep2 | RNAseq | SRX3503529 | (1) |
| CHE_96224 rep3 | RNAseq | SRX3503530 | (1) |
| CHE_94202 rep1 | RNAseq | SRX3503531 | (1) |
| CHE_94202 rep2 | RNAseq | SRX3503532 | (1) |
| CHE_94202 rep3 | RNAseq | SRX3503533 | (1) |
| GBR_JIW2 rep1 | RNAseq | SRX3503534 | (1) |
| GBR_JIW2 rep2 | RNAseq | SRX3503535 | (1) |
| GBR_JIW2 rep3 | RNAseq | SRX3503536 | (1) |
| ISR_7 rep1 | RNAseq | SRX17115170 | (2) |
| ISR_7 rep2 | RNAseq | SRX17115171 | (2) |
| ISR_7 rep3 | RNAseq | SRX17115172 | (2) |
| CHN_17_40 rep1 | RNAseq | SRX18362804 | (3) |
| CHN_17_40 rep2 | RNAseq | SRX18362805 | (3) |
| CHN_17_40 rep3 | RNAseq | SRX18362806 | (3) |

**Reference**

1. Praz CR, Menardo F, Robinson MD, Muller MC, Wicker T, Bourras S, et al. Non-parent

of Origin Expression of Numerous Effector Genes Indicates a Role of Gene Regulation in Host Adaption of the Hybrid Triticale Powdery Mildew Pathogen. Front Plant Sci. 2018;9:49.

1. Müller MC, Kunz L, Schudel S, Lawson AW, Kammerecker S, Isaksson J, et al. Ancient

variation of the AvrPm17 gene in powdery mildew limits the effectiveness of the introgressed rye Pm17 resistance gene in wheat. Proceedings of the National Academy of Sciences of the United States of America. 2022;119(30):e2108808119-e.

1. Kunz L, Sotiropoulos AG, Graf J, Razavi M, Keller B, Müller MC. The broad use of the Pm8 resistance gene in wheat resulted in hypermutation of the AvrPm8 gene in the powderymildew pathogen. BMC Biology. 2023;21(1):29.
